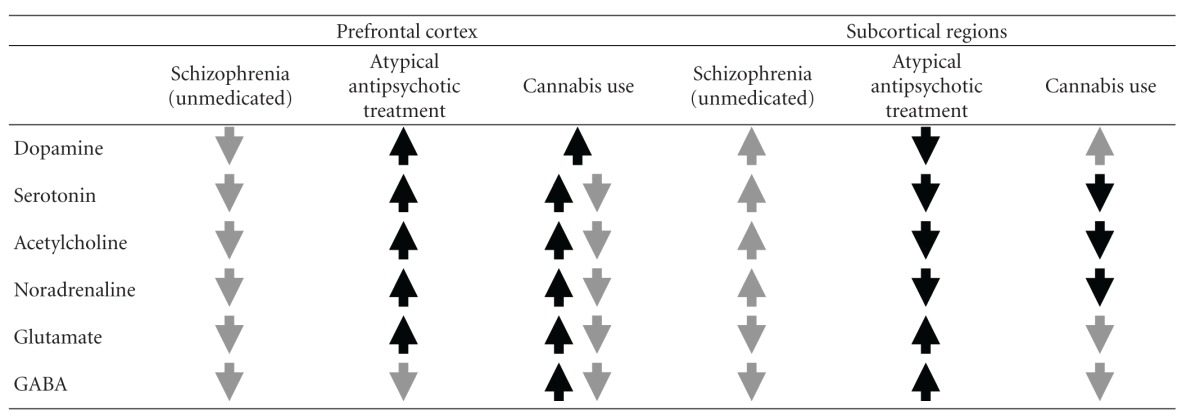# Cannabinoids for the Treatment of Schizophrenia? A Balanced Neurochemical Framework for Both Adverse and Therapeutic Effects of Cannabis Use

**DOI:** 10.1155/2011/501726

**Published:** 2010-07-27

**Authors:** Carissa M. Coulston, Michael Perdices, Antony F. Henderson, Gin S. Malhi

**Affiliations:** ^1^Discipline of Psychiatry, Sydney Medical School, University of Sydney, NSW 2006, Australia; ^2^Department of Psychiatry, CADE Clinic, Royal North Shore Hospital, St. Leonards, Sydney, NSW 2065, Australia; ^3^Department of Neurology, Royal North Shore Hospital, St. Leonards, Sydney, NSW 2065, Australia

## Abstract

Recent studies have found that cannabinoids may improve neuropsychological performance, ameliorate negative symptoms, and have antipsychotic properties for a subgroup of the schizophrenia population. These findings are in contrast to the longstanding history of adverse consequences of cannabis use, predominantly on the positive symptoms, and a balanced neurochemical basis for these opposing views is lacking. This paper details a review of the neurobiological substrates of schizophrenia and the neurochemical effects of cannabis use in the normal population, in both cortical (in particular prefrontal) and subcortical brain regions. The aim of this paper is to provide a holistic neurochemical framework in which to understand how cannabinoids may impair, or indeed, serve to ameliorate the positive and negative symptoms as well as cognitive impairment. Directions in which future research can proceed to resolve the discrepancies are briefly discussed.

## 1. Introduction

A body of literature over the past three decades has established cannabis use to be a significant risk factor for the onset and development of schizophrenia [1–5], with early onset of use (by age 15) believed to confer greater risk than later onset of use [6]. In a comprehensive meta-analysis of prospective and population-based studies by Moore et al. [7], an increased risk of psychosis was found in individuals who had ever used cannabis, and a dose-response effect was yielded in which greater risk applied to people who used cannabis most frequently. The authors could not, however, draw a definitive causal link between cannabis use and psychosis on grounds that the studies were observational in nature, and no robust evidence supports the view that early-age onset of cannabis use might be more harmful than later-age onset of use. The increased risk of psychosis in people using cannabis from a younger age could indicate greater cumulative exposure to cannabis rather than a sensitive period of exposure. 

The high prevalence of cannabis use in schizophrenia combined with observations that cannabis exacerbates the positive symptoms has driven research to investigate why cannabis is used so widely in this population. According to the self-medication hypothesis, there are beneficial effects of cannabis and cannabinoids on the symptoms of schizophrenia. Cross-sectional studies comparing users and nonusers have shown that cannabis use is associated with a reduction in negative symptoms [8–14]. Animal and human models have also suggested that cannabinoids possess antipsychotic properties [15–17]. One of these studies [15] involved a randomised, double-blind controlled trial in 42 patients with acute schizophrenia and found antipsychotic properties of cannabidiol (CBD) that were comparable to amisulpride. Other studies in which CBD has been administered to rats as well as healthy human volunteers have also demonstrated antipsychotic properties of CBD [16, 17]. 

These mixed findings on how cannabis or cannabinoids impact on the positive and negative symptoms of schizophrenia may in part be attributable to differences in methodology and research design. For example, studies have used different methods to measure cannabis exposure and assess outcome [7], and results are likely to be influenced by whether the studies were observational (prospective versus cross sectional) or experimental (direct drug administration by way of randomised controlled trials or other means). A range of other potential confounding factors are also likely to have contributed to the inconsistent results, particularly where observational designs were employed. 

From a neuropsychological perspective, a paucity of research exists. Cannabis use, in some studies of schizophrenia, has been found to be associated with worse neuropsychological performance [18–20]. Conversely, cannabis has been suggested to improve neuropsychological functioning [21–25]. Only one of these studies [18] involved a randomised, double-blind, placebo-controlled trial, in which the effects of i.v. ∆^9^-tetrahydrocannabinol (THC) on cognition were studied in 13 stabilised schizophrenia patients and 22 healthy controls, 30 minutes after drug administration. All participants administered THC relative to their placebo baseline performance demonstrated cognitive impairments in domains such as memory and attention, and the schizophrenia group performed more poorly than the control group. Five of the remaining studies [19, 21–25] were cross sectional in nature in which current or past cannabis users with schizophrenia were compared to non current users or those without a past history of use; and one study [20] performed a correlational analysis examining the relationship between level of cannabis use over the preceding year and cognition in persons with a psychotic illness.

Again, the inconsistencies in the findings between these neuropsychological studies are likely to be at least, in part, attributable to methodological variability between the studies, as well as methodological limitations within each study (for full review, see Coulston et al. [26]). For example, different methods were used to measure cannabis exposure, as per the studies detailed earlier which assessed the clinical symptoms. All seven neuropsychological studies used only a single index to classify cannabis use, which does not adequately reflect the variable and fluctuating level of cannabis use that may occur over time in the schizophrenia population. In three instances [19, 22, 23], cannabis use was defined solely with respect to abuse/dependence according to the Diagnostic and Statistical Manual of Mental Disorders, Fourth Edition (DSM-IV) [27]. In one study [18], only the acute effects of cannabis were examined, whilst in another study [21], only the residual, longer-term effects of cannabis were examined in participants who had been abstinent for at least 28 days. Such indices of cannabis use fail to consider factors such as recency and frequency of use (beyond the acute intoxication phase), which have been found to impact on cognition in the normal population [28]. 

Other limitations in several of these neuropsychological studies, some of which are also relevant to those studies mentioned earlier that focused on the associations between cannabis use and the positive/negative symptoms, include absence of drug screening procedures prior to assessment, restricted range of cognitive functions assessed, minimal or no control of important confounding variables such as current and past history of other substance use (including caffeine and nicotine), and a range of demographic and medical/psychiatric variables (e.g., gender, level of education, premorbid IQ, early-age onset of psychosis, duration of mental illness, medications). Furthermore, several of these studies did not include a control group which limits interpretation of their findings, because it cannot be determined if the cognitive performance of the cannabis users was frankly impaired (i.e., fell below the normal range of performance) or, was in fact within the normal range of performance.

One of the most comprehensive cross-sectional neuropsychological studies to date [29] was conducted by considering all the limitations and range of potential confounds detailed above. The investigators found that cannabis use was associated with enhanced cognitive functioning, predominantly in the areas of attention, processing speed, and executive functioning, domains which rely heavily on prefrontal cortical networks. 

Additional reasons for the discrepancies across the literature on the effects of cannabis use on symptomatology and cognition may pertain to the fact that separate and specialised groups of people with schizophrenia react differently to cannabis and other substances. Empirical literature indicates that systematic research on schizophrenia is difficult due to the disorder's heterogeneity and different diagnostic conceptualisations [30], which would imply that the neurochemical imbalances in different brain regions are not equal in all persons. As such, the schizophrenia population is deemed pharmacologically heterogenous [31], in which case the interaction between cannabinoids, neurotransmitters, and antipsychotic treatments is likely to vary between individuals. Subsequently, a balanced neurochemical framework to conceptualise *both* the adverse and potential therapeutic use of cannabinoids for respective groups of the population would be useful for two reasons; first, in light of the new emerging literature supporting a therapeutic benefit for all symptoms, and second, in the context of relatively minimal research that has been conducted in the field of neuropsychology. Further, positing a more balanced neurochemical framework that perhaps underpins an aspect of the core pathophysiology of schizophrenia allows a reconceptualisation of aetiology and treatment of this complex disorder. In particular, a balanced framework promotes awareness that there may not be a unidirectional method of treatment for all subgroups of the population, thereby providing a foundation for novel approaches to investigating the basis of this neuropsychiatric condition. 

This paper briefly outlines the neurochemical processes associated with schizophrenia and cannabis use in the normal population, with implications for the effects of cannabinoids on the positive and negative symptoms, as well as cognitive impairment.

## 2. Symptomatology in Schizophrenia and Neurotransmitter Systems

The effectiveness of conventional antipsychotic medications in alleviating positive symptoms is largely attributed to the blockade of dopamine transmission, especially in the mesolimbic system [32, 33]. Atypical antipsychotics are also believed to exert their therapeutic benefits on psychotic symptoms (in the same cerebral regions) via their antagonistic actions on serotonin, acetylcholine, and noradrenaline receptors [34–37], and via agonistic actions on GABA [35] and glutamate transmission [38]. This suggests that in the unmedicated state, the positive symptoms of schizophrenia are, at least in part, attributable to hyperactivation of dopamine, serotonin, acetylcholine, and noradrenaline, and hypoactivity of GABA and glutamate in the mesolimbic and other subcortical regions.

Moreover, reduced prefrontal dopamine activity is associated with exaggerated striatal dopaminergic activity [39], which is thought to account for the negative symptoms and cognitive impairment. Negative symptoms and cognitive impairments have also been associated with decreased prefrontal acetylcholine, serotonin, noradrenaline, and glutamate [39–47]. The therapeutic action of atypical antipsychotics involves facilitating prefrontal neurotransmission across all of these neurotransmitter systems, except GABA [48–53]. This suggests that in the unmedicated state, the negative symptoms and cognitive impairments of schizophrenia are, at least in part, attributable to hypoactivity of dopamine, serotonin, acetylcholine, noradrenaline, and glutamate in the prefrontal region.

With respect to GABA, although direct enhancement of prefrontal activity would be a potential treatment for schizophrenia [54], atypical antipsychotics tend to exert their effect in the prefrontal cortex by inhibiting release of GABA from interneurons [55]. This subsequently enhances prefrontal neurotransmitter activity. Dopamine and serotonin signalling, associated with antipsychotics, may therefore be responsible for the inhibition of GABAergic currents, and subsequent increase in prefrontal activity [56, 57].

## 3. The Endocannabinoid System

Cannabinoids exert their effect by binding to specific *endogenous cannabinoid receptors *[58] known as CB1 and CB2, which are located in the CNS and peripherally in the spleen and immune cells [59, 60]. The CB1 receptors are widely distributed in the brain including the cerebral cortex (especially the frontal and medial temporal lobes), limbic areas, basal ganglia, ventral tegmental area, thalamus, hypothalamus, cerebellum, and brainstem [58, 61–65]. 

CB1 receptors reside within the lipid membrane of the presynaptic neuron terminals, are modulated by the postsynaptic release of endocannabinoids (namely anandamide and 2-arachidonoyl glycerol), and are also influenced by exogenously consumed cannabinoids. CB1 receptors act as neuromodulators through coupling with intracellular G-proteins controlling cyclic adenosine monophosphate (c-AMP) formation and Ca2+ and K+ transport. In this respect, the cannabinoid system has important interactions with several neurotransmitter systems.

Cannabinoids augment potassium stimulated striatal dopamine efflux and subsequently dopamine release in the mesolimbic system (especially the nucleus accumbens), medial prefrontal cortex, midbrain regions, and substantia nigra [58, 66–74]. THC has been shown to facilitate stimulation of dopamine release in the medial forebrain bundle of the mesolimbic system at dose ranges pharmacologically relevant to human recreational use [68]. As the positive symptoms of schizophrenia are related to elevated dopamine release, particularly in the mesolimbic system, cannabinoids have long been implicated in worsening the positive symptoms of schizophrenia [73, 75, 76]. 

In contrast to the effect of cannabinoids on dopamine release in the mesolimbic system, significant evidence exists for the CB1 receptor mechanism to play an important role in “dampening” neuroexcitability [59, 60, 77]. It has been demonstrated that in the cerebral cortex, serotonin activity is inhibited by cannabinoid receptor agonists (e.g., WIN 55,212 and CP-55,940) [78]. Conversely, studies employing CB1 receptor antagonists (e.g., SR 141716A) have demonstrated increases in serotonin in various brain regions including the medial prefrontal cortex and nucleus accumbens [79]. 

Cannabinoids have been demonstrated to decrease acetylcholine release in the medial prefrontal cortex, hippocampus, and striatum [80–82]. Likewise, the CB1 receptor antagonist (SR 141716A) has resulted in an increase in acetylcholine in the medial prefrontal cortex and hippocampus [79, 83]. 

Noradrenaline activity in various areas of the brain including the hippocampus, cerebellum, hypothalamus, and cerebral cortex, has been inhibited by the cannabinoid receptor agonist (WIN 55,212) [84]. This inhibition was subsequently attenuated by the cannabinoid receptor antagonist (SR 141716). Cannabinoid-inhibited release of noradrenaline has also been demonstrated in the hypothalamus and striatum [60, 85–87]. 

The CB1 receptor has been demonstrated to be present in high concentration at presynaptic terminals of glutaminergic synapses in the hippocampus and other areas of the brain [88]. In general, activation of the CB1 receptor has been shown to inhibit glutamate release in the cerebellum, hippocampus, prefrontal cortex, and substantia nigra [77, 78, 89–91]. 

CB1 receptor mediated decreases in GABA has also been demonstrated in brain regions including the prefrontal cortex [78, 88, 92, 93], hippocampus [90, 94, 95], and subcortical regions including the basal ganglia [96–100].

## 4. Cannabinoids and Prefrontal Neurotransmission

Despite the general consensus that cannabinoids inhibit all major neurotransmitters (except dopamine) in various brain regions including the prefrontal cortex, a number of studies have supported the converse notion that cannabinoids in fact stimulate neurotransmission release in the prefrontal cortex [88, 92, 93]. 

An example of how neurotransmission of dopamine could be theoretically enhanced by cannabinoids in the prefrontal cortex is related to CB1 receptors being preferentially located on GABAergic interneurons in areas including the cerebral cortex, ventral tegmental area, and hippocampus [92]. Cannabinoids such as THC decrease the excitability of GABA-ergic interneurons, and therefore, given GABA is the major inhibitory neurotransmitter in the CNS, disinhibition of dopamine and glutamate follows. Consequently, increases in the outflow of neurotransmission parallel the decrease in extracellular GABA.

As another example of how neurotransmission is theoretically enhanced by cannabinoids in the prefrontal cortex, studies have demonstrated that increased dopamine neurotransmission in the mesolimbic projections from the nucleus accumbens triggers an inhibition of GABA-ergic efferents to the basal forebrain. The process of inhibiting the release of GABA, in turn, decreases the inhibition of other neurotransmitters such as dopamine, acetylcholine, noradrenaline, and glutamate. A specific example of this process has been postulated where dopamine's direct inhibitory effect on both glutamate and GABA-ergic activity leads to an increased excitability of cholinergic flow [46, 101]. 

Proceeding from these lines of reasoning, studies have demonstrated that in the prefrontal region, cannabinoids mediate increases in noradrenaline [102], acetylcholine [103, 104], and glutamate [92, 105]. With respect to serotonin and GABA, studies have demonstrated that cannabinoids mediate the inhibition of *reuptake *in cortical areas, which leads to their increase [106].

## 5. The Effects of Cannabis on Cognition and the Positive/Negative Symptoms of Schizophrenia

Table 1 presents a summary of the information discussed thus far. The grey arrows in the “Schizophrenia” columns represent the directions of neurotransmitter release in the unmedicated state of the disorder in both the prefrontal cortex and subcortical regions. As discussed above, the negative symptoms and cognitive impairments are generally conceptualised in terms of decreased neurotransmission of all six major systems in the prefrontal cortex, whilst the positive symptoms are generally thought to arise because of increased neurotransmission of dopamine, serotonin, acetylcholine and noradrenaline systems, and decreased neurotransmission of glutamate and GABA systems in subcortical regions.

The black arrows in the “Atypical antipsychotic treatment” columns represent how these medications are purported to ameliorate the symptoms and cognitive impairments of schizophrenia. Specifically, atypical antipsychotics are postulated to ameliorate the negative symptoms and cognitive impairment by stimulating neurotransmission in the prefrontal cortex (of all neurotransmitters except GABA), and are thought to ameliorate the positive symptoms by inhibiting dopamine, serotonin, acetylcholine, and noradrenaline, and by increasing glutamate and GABA in subcortical regions.

Of pertinent interest to this review is the theoretical amelioration and/or exacerbation of the symptoms and cognitive impairments of schizophrenia that cannabis use may produce. As Table 1 shows, cannabis in the normal population leads to excitation of dopamine in the prefrontal cortex. However, what remains relatively less certain is whether cannabis leads to excitation or inhibition of the other five major neurotransmitters in the prefrontal cortex (i.e., serotonin, acetylcholine, noradrenaline, glutamate, and GABA), given that evidence for both modes of action have been presented. Hence, the postulated effect of cannabis on these five systems is indicated by both grey and black arrows.

Consequently, looking at the “Schizophrenia (unmedicated)” and “Cannabis use” columns in conjunction, Table 1 shows that although cannabis has the potential to exacerbate the negative symptoms and cognitive impairments of schizophrenia via contributing further to the “hypofrontal” nature of the disorder, cannabis also has a therapeutic potential by facilitating neurotransmission (at least in the dopaminergic system) in the prefrontal cortex. In line with the latter perspective, research has demonstrated enhanced cognitive performance in schizophrenia with comorbid cannabis use [21–25, 29], which may be attributable to stimulation of prefrontal neurotransmission.

In subcortical regions, Table 1 shows that cannabis use is associated with excitation of dopamine and inhibition of all the other five major neurotransmitters (i.e., serotonin, noradrenaline, acetylcholine, glutamate, and GABA). Hence, cannabis has the potential to counteract increased levels of serotonin, acetylcholine, and noradrenaline that occur in subcortical regions in untreated schizophrenia and thus, provide an antipsychotic therapeutic effect. However, excitation of dopamine and inhibition of glutamate and GABA in subcortical regions produced by cannabis use may augment the dysregulation that occurs in these three systems in untreated schizophrenia, and possibly exacerbate the positive symptoms.

## 6. Summary and Conclusion

Cannabis use in the schizophrenia population has been shown to worsen the prognosis and increase the burden of the disorder. However, evidence exists for a subgroup of the population to suggest that cannabinoids have therapeutic effects on the negative and positive symptoms, as well as cognitive impairments. Although this evidence is not conclusive and requires further research and replication, a more comprehensive and broad-based neurochemical framework has been presented in this paper, offering an explanation for the potentially therapeutic effects of cannabinoids in addition to its adverse effects.

Whilst the neurochemical effects of cannabinoids are complex, cannabinoids appear to have at least in part, a “restorative” effect on neurotransmitter dysfunctions in schizophrenia, which may underpin the biological substrate of the therapeutic effects cannabis has been demonstrated to have in recent studies.

In the context of recent research by Coulston et al. [29], future studies need to establish which subgroups of schizophrenia most benefit from cannabinoids as a putative “treatment”; which subgroups demonstrate nil or minimal exacerbation of positive symptoms in the context of cannabis use, or indeed, which subgroups may experience antipsychotic effects of cannabis; how such a treatment may complement or interact with standard antipsychotics and other psychiatric treatments; what constitutes an effective quantity or dose of cannabinoids to exert a beneficial impact on cognition; what precise frequency of administration would be required; how long the effects of cannabinoids last on cognition following a recent dose; how such a treatment may generalise to other domains of real-life functioning such as employment and social settings (and in particular, cognitive processes required to function well in these settings); and whether such a treatment can be generalised to older age groups. Methods by which future research could proceed to address these questions include randomised, double-blind, controlled drug trials.

Clearly there are many questions that need to be addressed and the use of further randomised, double-blind studies is necessary to establish the effects of cannabis across the domains of cognition, mood, and mentation. However, given the prevalence of cannabis use and its integral role in the clinical manifestation of psychosis, this is an area for future research, and reconceptualising our neurochemical understanding is perhaps a useful first step. In this context, the consideration, appraisal, and rigorous testing of novel models are necessary, and are likely to advance our understanding.

## Figures and Tables

**Table 1:** Directions of neurotransmitter release in the prefrontal cortex and subcortical regions.